# Survival outcomes in men with a positive family history of prostate cancer: a registry based study

**DOI:** 10.1186/s12885-020-07174-9

**Published:** 2020-09-18

**Authors:** Mann Ang, Martin Borg, Michael E. O’Callaghan, for the South Australian Prostate Cancer Clinical Outcomes Collaborative (SA-PCCOC)

**Affiliations:** 1 The University of Adelaide, Adelaide, South Australia Australia; 2 Central Adelaide Local Health Network, Adelaide, South Australia Australia; 3 South Australian Prostate Cancer Clinical Outcomes Collaborative, Adelaide, South Australia Australia; 4 Adelaide Radiotherapy Centre, GenesisCare, Adelaide, South Australia Australia; 5 Discipline of Medicine, Freemasons Foundation Centre for Men’s Health, University of Adelaide, Adelaide, South Australia Australia; 6 Flinders Medical Centre, Urology Unit, Flinders University, Flinders Drive, Bedford Park, South Australia SA 5042 Australia

**Keywords:** Prostate Cancer, Family history, Survival, Outcomes, Genetics

## Abstract

**Background:**

To investigate the correlation between family history of prostate cancer (PCa) and survival (overall and cancer specific) in patients undergoing treatment for PCa.

**Methods:**

ine thousand four hundred fifty-nine patients with PCa were extracted from the South Australian Prostate Cancer Clinical Outcomes Collaborative (SA-PCCOC) database. Diagnosis occurred after 1998 and treatment before 2014. Cox proportional-hazards modeling was used to assess the effect of family history on overall survival after adjustment for confounders (age at diagnosis, NCCN risk category and year of treatment), and with stratification by primary treatment group. Competing risks regression modelling was used to assess PCa specific mortality.

**Results:**

Men with a positive family history of PCa appear to have a lower Gleason score at the time of diagnosis (50% with Gleason < 7, compared to 39% in those without family history) and be diagnosed at a lower age (64 vs 69). Men with a positive family history of PCa appear to have better overall survival outcomes (*p* < 0.001, log rank test). In analysis adjusting for age at diagnosis, NCCN risk category and year of treatment, family history remained a significant factor when modelling overall survival (HR 0.72 95% CI 0.55–0.95, *p* = 0.021).

There were no significant differences in treatment subgroups of radical prostatectomy (*p* = 0.7) and radiotherapy (0.054).

**Conclusion:**

Men with a positive family history of PCa appear to have better overall survival outcomes. This better survival may represent lead time bias and early initiation of PSA screening. Family history of PCa was not associated with different survival outcomes in men who were treated with either radical prostatectomy or radiotherapy.

## Background

Having a family history of prostate cancer is a known risk factor for developing prostate cancer. Having a single first-degree relative with prostate cancer increases the risk of developing prostate cancer by a factor of 2.1–2.8. Having two affected relatives increases the risk 3.5- fold [1].

Whilst most cases of prostate cancer are associated with somatic mutations, inherited gene changes can increase the risk of development of prostate cancer (for review see [2]). Examples of such genes include tumour suppressor genes, BRCA1 and BRCA2 [3].

Family history has been examined in the context of prostate cancer and overall survival following PSA testing [4]. A number of studies describe whether a positive family history is a prognostic factor following the diagnosis of prostate cancer. Several studies have been published worldwide, mainly examining the relationship between a positive family history and clinical outcomes after radical prostatectomy [5–7]. No studies have been published to date in Australia.

The purpose of this study is to examine whether having a family history of prostate cancer affects clinical outcomes in an Australian cohort. Outcomes of interest are overall survival and prostate cancer specific mortality. Subgroup analysis by treatment group (radical prostatectomy and radiation therapy) will be conducted.

## Methods

### Patients

The South Australia Prostate Cancer Clinical Outcomes Collaborative (SA-PCCOC) database enrolls men with prostate cancer diagnosed in public hospitals and collaborating private institutions in South Australia. It was established in 1998 and recruits men with a histologic diagnosis of prostate cancer including by prostate biopsy and also incidental findings after treatment for other conditions (e.g. transurethral resection of the prostate for treatment of lower urinary tract symptoms).

Men with diagnosis post 1st January 1998 and treatment prior to 1st June 2014 were included. The following data were extracted: age at diagnosis, reason for referral to a specialist for prostate biopsy, PSA measurement at diagnosis, family history of prostate cancer, clinical staging (based on digital rectal examination, laboratory investigations and imaging at the time of diagnosis), Gleason score at the time of diagnosis, primary treatment, treatment year and survival time (the time between date of diagnosis and date of death or censor). A modified (low, intermediate and high risk categories) NCCN risk classification was used to assign risk groups to men based on their PSA levels at diagnosis, diagnostic PSA and Gleason score. This modified score is used for Australian population level studies [8, 9]. Family history was gathered from medical notes and included binary responses relating to grandfather, father, uncle, child and grandchild.

### Analysis

Demographic data were tabulated and comparisons made between groups using the Chi squared test (categorical variables) or ANOVA (continuous variables). Overall survival was compared in patients with and without a family history of prostate cancer after diagnosis as well as by primary treatment modality (post radical prostatectomy or post radiotherapy). Survival was plotted using Kaplan-Meier curves and groups compared using a log-rank test. Cox proportional hazards modelling was used to assess overall survival and the effect of a positive family history on survival after adjustment for confounders (age at diagnosis, NCCN [10] staging and year of treatment [continuous variable]). Cumulative incidence plots were used to display death by prostate cancer and other causes. Cause of death data was taken from the South Australian Births, Deaths and Marriages Registry and also the South Australian Cancer Registry. Prostate cancer was attributed as a cause of death where this was documented as a primary or significant contributing cause by the doctor completing the death certificate. Fine and Grey modelling was used to predict prostate cancer specific mortality with adjustment in multivariable analysis as described above. All analyses were conducted in R, and *p* < 0.05 was taken to indicate statistical significance.

The Southern Adelaide Clinical Human Research Ethics Committee reviewed and approved the SA-PCCOC database, including analysis of data without identifiers, such as this study.

## Results

### Patient demographics

9459 men were identified after applying exclusion criteria. Patient demographics are shown in Table 1. Men with a positive family history of prostate cancer appear to have a lower Gleason score at the time of diagnosis and be diagnosed at a lower age (*p* < 0.05).

**Table 1:** – Demographics

Variable		Family History		
		No	Yes	***p***
n		8801	658	
Age at diagnosis (median (IQR))		69[62,76]	63[57,69]	< 0.001
PSA at diagnosis (%)	1. < 4	624 (7.1)	65 (9.9)	< 0.001
	2. 4–10	3156 (35.9)	333 (50.6)	
	3. 10–20	1644 (18.7)	119 (18.1)	
	4. > 20	1395 (15.9)	59 (9.0)	
	5. Missing	1982 (22.5)	82 (12.5)	
Gleason Score at biopsy (%)	< 7	3465 (39.4)	336 (51.1)	< 0.001
	3 + 4	1805 (20.5)	152 (23.1)	
	4 + 3	1062 (12.1)	73 (11.1)	
	8	799 (9.0)	39 (6.1)	
	8+	678 (7.6)	20 (3.1)	
	Missing	982 (11.2)	36 (5.5)	
NCCN Risk classification (%)	High	519 (12.2)	32 (7.3)	0.005
	Intermediate	430 (10.1)	38 (8.7)	
	Low	3313 (77.7)	366 (83.9)	
Reason for Biopsy (%)	1. Elevated PSA	4626 (52.6)	451 (68.5)	< 0.001
	2. Prostate Symptoms	1770 (20.1)	96 (14.6)	
	3. Other Symptoms	520 (5.9)	76 (11.6)	
	4. Not Known	1885 (21.4)	35 (5.3)	
Treatment year (%)	< 2005	1399 (20.0)	83 (13.8)	< 0.001
	> = 2005	5606 (80.0)	518 (86.2)	
Treatment group (%)	Hormones	1113 (12.6)	36 (5.5)	< 0.001
	Other	2234 (25.4)	89 (13.5)	
	Not yet classified	18 (0.2)	1 (0.2)	
	Radical Prostatectomy	2949 (33.5)	293 (44.5)	
	Radiation therapy	2487 (28.3)	239 (36.3)	
M stage at diagnosis (%)	M0	1263 (96.7)	118 (99.2)	0.229
	M1	43 (3.3)	1 (0.8)	
N stage at diagnosis (%)	N0	836 (97.2)	87 (98.9)	0.566
	N1	24 (2.8)	1 (1.1)	
Family members reported to have a positive history				
Grandfather (%)	No	7811 (100.0)	634 (96.4)	< 0.001
	Yes	0 (0.0)	24 (3.6)	
Parent (%)	No	7811 (100.0)	372 (56.5)	< 0.001
	Yes	0 (0.0)	286 (43.5)	
Sibling (%)	No	7811 (100.0)	438 (66.6)	< 0.001
	Yes	0 (0.0)	220 (33.4)	
Child (%)	No	7811 (100.0)	653 (99.2)	< 0.001
	Yes	0 (0.0)	5 (0.8)	
Grandchild (%)	No	7811 (100.0)	658 (100.0)	NA
Uncle (%)	No	7811 (100.0)	606 (92.1)	< 0.001
	Yes	0 (0.0)	52 (7.9)	
Cause of death (%)	Not prostate cancer	1482 (57.8)	52 (55.9)	0.795
	Prostate cancer	1081 (42.2)	41 (44.1)	

There was a significant difference in the reasons for referral for biopsy with those having a positive family history being more likely to report having an elevated PSA (68.5% vs 52.6%) and less likely to have prostate related symptoms (14.6% vs 20.1%). The most common relation in those reporting to have a positive family history was parent (56.5%) followed by sibling (33.4%).No men reported having a grandchild with prostate cancer.

There are significant treatment differences between the group of men with and without family history of prostate cancer e.g. hormones 5.5% vs 12.6%, radical prostatectomy 44.5% vs 33.5%, radiation therapy 36.3% vs 28.3% (all *p* < 0.05). The ‘other’ treatment group included those that could not be classified as one of the above active treatment groups, and included those on observation e.g. watchful waiting or active surveillance.

### Overall survival time after diagnosis

**Fig. 1:**
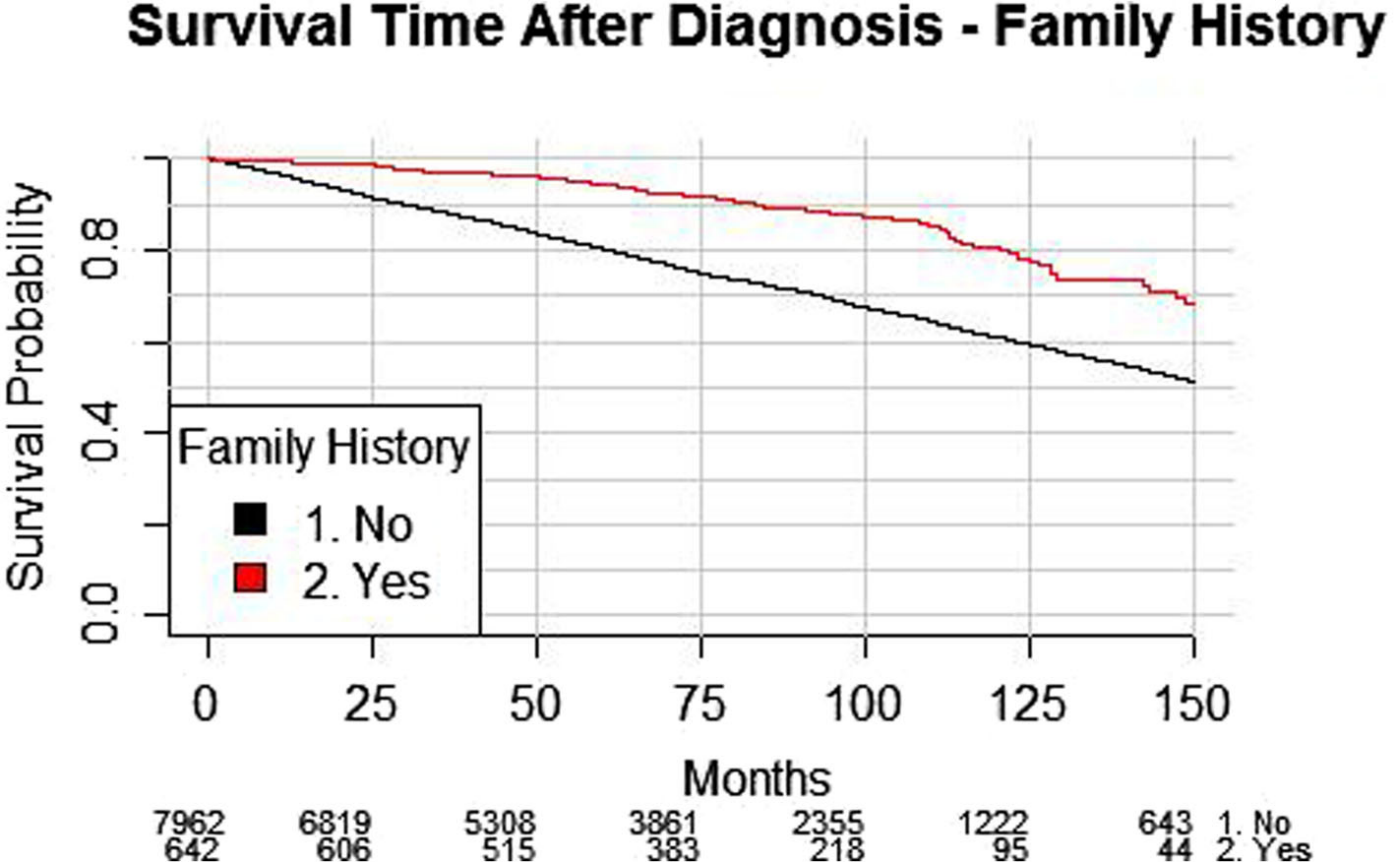
– Survival post diagnosis by family history (*p* < 0.001)

In a univariable Log-rank test, men with a family history of prostate cancer appear to have better survival outcomes (*p* < 0.001, see Fig. 1). In analysis adjusting for age at diagnosis, NCCN risk category and year of treatment, family positive history remained a significant factor when modelling overall survival (Supplementary Table 1, HR 0.74, 95% CI 0.57–0.97, *p* = 0.027).

**Fig. 2:**
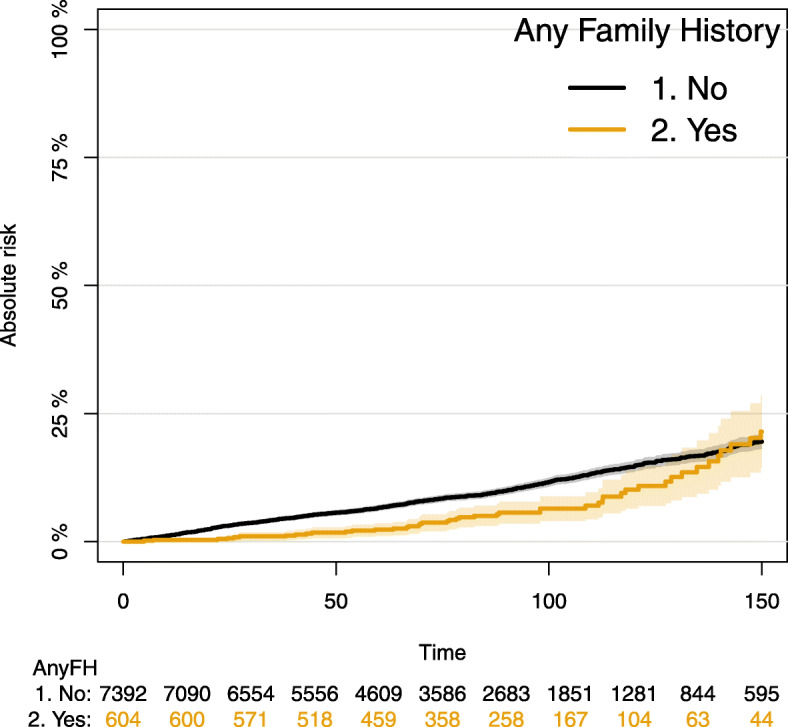
Survival post treatment and family history. **a** – Radical prostatectomy (*p* = 0.70), **b** – Radiation therapy (*p* = 0.054)

In a univariable Log-rank test, men with a family history of prostate cancer who were treated with radical prostatectomy did not appear to have different survival outcomes (*p* = 0.70, see Fig. 2). This result was unchanged when the cohort was further restricted to those aged 50–70 years at diagnosis with low or intermediate risk disease. In analysis adjusting for age at diagnosis, NCCN risk category and year of treatment, family history was not a significant factor when modelling overall survival (supplementary Table 3, HR 0.89, 95% CI 0.47–1.66, *p* = 0.70).

In a univariable Log-rank test, men with a family history of prostate cancer who were treated with radiation therapy did not appear to have different survival outcomes (*p* = 0.054, see Fig. 2b). This result was unchanged when the cohort was further restricted to those aged 50–70 years at diagnosis with low or intermediate risk disease. In analysis adjusting for age at diagnosis, NCCN risk category and year of treatment, family history was not a significant factor when modelling overall survival, in men treated with XRT (supplementary Table 4, HR 0.65, 95% CI 0.41–1.0, *p* = 0.054).

Age at treatment was associated with overall survival. (radical prostatectomy – HR 1.06, 95% CI 1.03–1.10, *p* < 0.001; radiation therapy HR 1.06, 95% CI 1.05–1.08, *p* < 0.001; supplementary Tables 3 and 4).

**Fig. 3:**
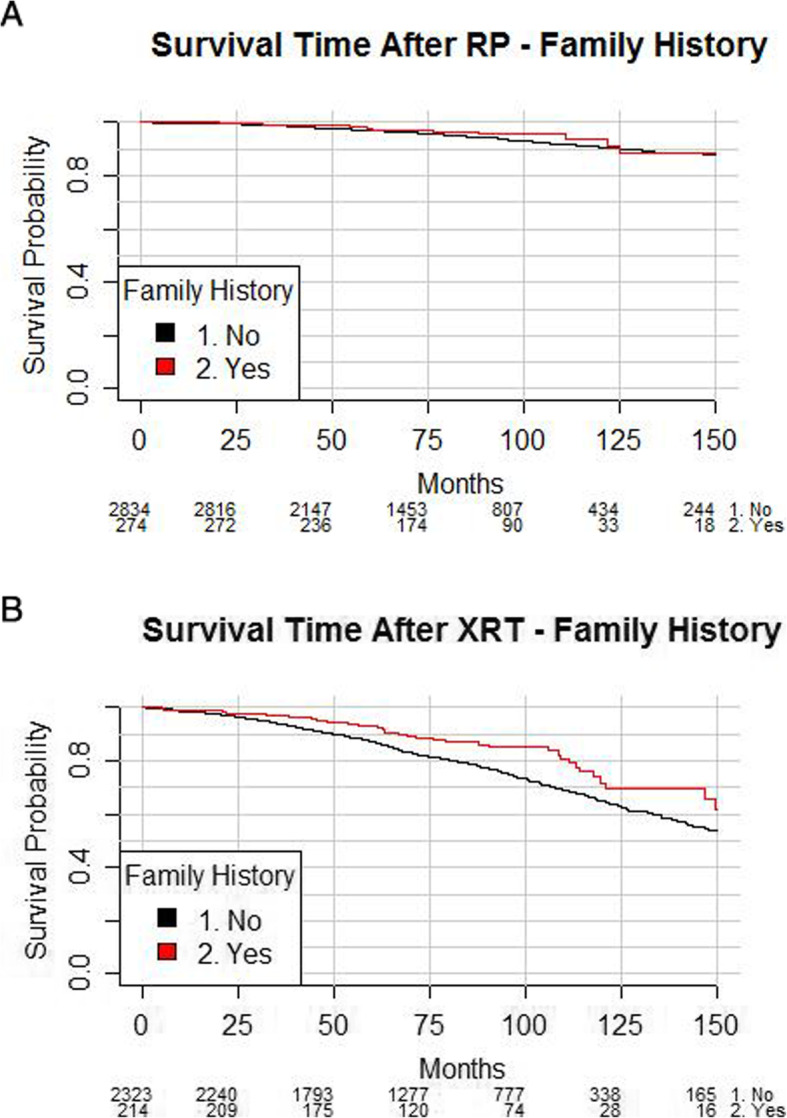
– prostate cancer specific mortality by family history (*p* > 0.05)

No differences in prostate cancer specific mortality were detected between men in this cohort with a positive family history of prostate cancer and those without (Fig. 3, *p* > 0.05, Supplementary Table 2). Subgroup analysis of prostate cancer specific mortality by treatment group was not undertaken due to low event numbers.

## Discussion

In our cohort of 9459 men with prostate cancer, a positive family history of the disease was reported in 658 (6.9%). Having a positive family history was associated with significantly longer overall survival; however it was not associated with any change in prostate cancer specific mortality.

Heritability of prostate cancer is estimated to be 57% (95% CI 51–63, [11]) with 100 genetic loci thought to contribute to one third of the genetic component of this disease [12, 13]. While this aspect of the disease – incidence- has been studied extensively there is less literature relating to the impact of heritability and genetics on outcomes of men who are diagnosed. The overall survival advantage observed in our cohort may be related to the fact that patients with a positive family history for prostate cancer appeared to be diagnosed at a younger age and as a result of elevated PSA measurements as opposed to symptomatic presentations. This could be interpreted to mean that these patients are screened more aggressively and from an earlier age. In the literature, Lee et al. [6] have also observed improved disease-free survival in patients reporting a positive family history of prostate cancer compared to those without a family history. This was thought to be related to the earlier age at diagnosis and improved pathologic features in the group with a positive family history rather than to any true biologic differences in their cancers.

Better survival may represent lead time bias and early initiation of PSA screening. The current clinical practice guidelines on PSA testing in Australia do not recommend a national PSA screening program [14]. However, recommendations in the guidelines include offering PSA testing every 2 years for men ages between 40 and 69 in men with a family history of prostate after being informed of the benefits and harms of testing. This recommendation contrasts to men without a positive family history where testing, if it occurs, is recommended to commence at age 50 years. Earlier studies such as Gronberg et al. [15] have shown no difference in overall-survival or prostate cancer specific survival in patients with and without a positive family history. The discrepancy with our work may be explained by the small sample size (302) and historic nature of the cohort (diagnosed between 1958 and 1990). During this time period there have been numerous changes to prostate cancer screening and treatment meaning that this work may not be generalizable to the clinical setting today.

Men with a family history of prostate cancer who were treated with radical prostatectomy did not appear to have different survival outcomes compared with men who did not have a family history. The results of our study are consistent with previous findings such as Bauer et al. [16], Brath et al. [17] and Bagshaw et al. [18], although contradict those reported by Kupelian et al. [19].

To date, there have been few published studies examining survival outcomes following radiation therapy and the impact of family history of prostate cancer [20]. The results of our study suggest that there is no survival difference in men receiving radiation therapy, comparing those with and without a positive family history, which is consistent with literature to date. While some cases of prostate cancer which are diagnosed in the setting of a positive family history are more aggressive, there is also an increase in low risk disease, presumably as a result of increased surveillance and investigation [21]. This later case may account for the results we observe in both surgical and radiotherapy treated cases.

### Limitations

Our study has limitations including not having any data for family history of other cancers (e.g. pancreatic, breast, ovarian). These cancers may also be relevant in investigating genes that confer lifetime risks of prostate cancer, such as BRCA1 and BRCA2 [22–24]. The demographic and clinical differences between the groups may confound the observations made. While we have used multivariable modelling to adjust for potential confounders, the effect of residual confounding or unmeasured confounding cannot be eliminated, and may also be limited where data is missing. We note also that this cohort is drawn from an Australian cohort and country specific PSA screening and treatment practices may limit generalisability to other settings. Sample size also presents a limitation of the current work. An example of this is in the group of men treated with radiotherapy where the hazards ratio is similar in the subgroup compared with the overall cohort, but the estimate is less precise.

## Conclusion

In summary, men with a family history of prostate cancer appear to have better overall survival outcomes. This group of men also appear to have a lower Gleason score at the time of diagnosis and be diagnosed at a lower age. Family history of prostate cancer was not associated with different survival outcomes in men who were treated with either radical prostatectomy or radiotherapy.

## Supplementary information

**Additional file 1 Supplementary Table 1** – Cox proportional hazards model – overall survival. **Supplementary Table 2** – Fine and Grey model predicting prostate cancer specific mortality. **Supplementary Table 3** - Cox proportional hazards model – overall survival – Radical prostatectomy. **Supplementary Table 4**- Cox proportional hazards model – overall survival – Radiation therapy

## Data Availability

Data is available upon request, but access may be subject to ethics and institutional governance approvals.

## Abbreviations

SA-PCCOC: South Australian Prostate cancer clinical outcomes collaborative
PSA: Prostate specific antigen
HR: Hazards ratio
CI: Confidence interval
PCa: Prostate cancer
NCCN: National comprehensive cancer network
