# Supplementary material for: Survival outcomes in men with a positive family history of prostate cancer: a registry based study
Source: BMC Cancer. 2020 Sep 18;20:894. doi: 10.1186/s12885-020-07174-9 (PMC7499864; doi:10.1186/s12885-020-07174-9)
Supplement: Supplementary file 1 — Additional file 1 Supplementary Table 1 – Cox proportional hazards model – overall survival. Supplementary Table 2 – Fine and Grey model predicting prostate cancer specific mortality. Supplementary Table 3 - Cox proportional hazards model – overall survival – Radical prostatectomy. Supplementary Table 4- Cox proportional hazards model – overall survival – Radiation therapy [file 12885_2020_7174_MOESM1_ESM.docx]

# Supporting material

Supplementary Table 1 – Cox proportional hazards model – overall survival

| **Variables** | **Hazards Ratio** | **Lower 95% CI** | **Lower 95% Upper CI** | **P Value** |
| --- | --- | --- | --- | --- |
| **Family History - No** | - | - | - | - |
| **Family History - Yes** | **0.742** | **0.57** | **0.966** | **0.027** |
| **Age at diagnosis** | **1.091** | **1.083** | **1.099** | **<0.001** |
| **NCCN - High** | - | - | - | - |
| **NCCN - Intermediate** | **0.474** | **0.391** | **0.575** | **<0.001** |
| **NCCN - Low** | **0.412** | **0.355** | **0.477** | **<0.001** |
| **Treatment year** | **0.967** | **0.951** | **0.983** | **<0.001** |

Supplementary Table 2 – Fine and Grey model predicting prostate cancer specific mortality

| **Variables** | **Sub-Hazards Ratio** | **Lower 95% CI** | **Lower 95% Upper CI** | **P Value** |
| --- | --- | --- | --- | --- |
| **Family History - No** | - | - | - | - |
| **Family History - Yes** | 0.748 | 0.506 | 1.106 | 0.15 |
| **Age at diagnosis** | 1.052 | 1.04 | 1.064 | <0.001 |
| **NCCN - High** | 4.895 | 3.895 | 6.153 | <0.001 |
| **NCCN - Intermediate** | 1.828 | 1.408 | 2.372 | <0.001 |
| **NCCN - Low** | **-** | **-** | **-** | **-** |
| **Treatment year** | 0.952 | 0.931 | 0.974 | <0.001 |

Supplementary Table 3 - Cox proportional hazards model – overall survival – Radical prostatectomy

| **Variables** | **Hazards Ratio** | **Lower 95% CI** | **Lower 95% Upper CI** | **P Value** |
| --- | --- | --- | --- | --- |
| **Family History - No** | - | - | - | - |
| **Family History - Yes** | 0.885 | 0.472 | 1.657 | 0.703 |
| **Age at diagnosis** | 1.064 | 1.031 | 1.098 | <0.001 |
| **NCCN - High** | - | - | - | - |
| **NCCN - Intermediate** | 1.029 | 0.230 | 4.613 | 0.97 |
| **NCCN - Low** | 0.860 | 0.212 | 3.495 | 0.833 |
| **Treatment year** | 0.983 | 0.923 | 1.046 | 0.585 |

Supplementary Table 4- Cox proportional hazards model – overall survival – Radiation therapy

| **Variables** | **Hazards Ratio** | **Lower 95% CI** | **Lower 95% Upper CI** | **P Value** |
| --- | --- | --- | --- | --- |
| **Family History - No** | - | - | - | - |
| **Family History - Yes** | 0.645 | 0.412 | 1.008 | 0.054 |
| **Age at diagnosis** | 1.063 | 1.046 | 1.080 | <0.001 |
| **NCCN - High** | - | - | - | - |
| **NCCN - Intermediate** | 0.626 | 0.448 | 0.875 | 0.006 |
| **NCCN - Low** | 0.573 | 0.433 | 0.758 | <0.001 |
| **Treatment year** | 0.973 | 0.944 | 1.002 | 0.072 |
